# Inherited bone marrow failure syndromes: phenotype as a tool for early diagnostic suspicion at a major reference center in Mexico

**DOI:** 10.3389/fgene.2023.1293929

**Published:** 2024-01-24

**Authors:** Paula Leal-Anaya, Tamara N. Kimball, Ana Lucia Yanez-Felix, Moisés Ó. Fiesco-Roa, Benilde García-de Teresa, Angélica Monsiváis, Rocío Juárez-Velázquez, Esther Lieberman, Camilo Villarroel, Emiy Yokoyama, Liliana Fernández-Hernández, Anet Rivera-Osorio, David Sosa, Maria Magdalena Ortiz Sandoval, Norma López-Santiago, Sara Frías, Victoria del Castillo, Alfredo Rodríguez

**Affiliations:** ^1^ Departamento de Medicina Genómica y Toxicología Ambiental, Universidad Nacional Autónoma de México, México City, Mexico; ^2^ Laboratorio de Falla Medular y Carcinogénesis, Instituto Nacional de Pediatría, México City, Mexico; ^3^ Posgrado en Ciencias Biomédicas, Universidad Nacional Autónoma de México, México City, Mexico; ^4^ Departamento de Genética Humana, Instituto Nacional de Pediatría, México City, Mexico; ^5^ Programa de Maestría y Doctorado en Ciencias Médicas, Odontológicas y de la Salud, Universidad Nacional Autónoma de México, México City, Mexico; ^6^ Laboratorio de Citogenética, Instituto Nacional de Pediatría, México City, Mexico; ^7^ Departamento de Hematología, Instituto Nacional de Pediatría, México City, Mexico; ^8^ Laboratorio de Genética y Cáncer, Instituto Nacional de Pediatría, México City, Mexico; ^9^ Laboratorio de Biología Molecular, Instituto Nacional de Pediatría, México City, Mexico; ^10^ Laboratorio de Análisis Genéticos Especializados México (LAGEM), México City, Mexico; ^11^ Servicio de Hematologia Oncologia Pediatrica Hospital Civil de Guadalajara JIM, Guadalajara, México

**Keywords:** inherited bone marrow failure syndrome, dyskeratosis congenita, Diamond–Blackfan anemia, Shwachman–Diamond syndrome, thrombocytopenia with absent radii, severe congenital neutropenia

## Abstract

**Introduction:** The inherited bone marrow failure syndromes (IBMFSs) are a group of rare disorders characterized by bone marrow failure (BMF), physical abnormalities, and an increased risk of neoplasia. The National Institute of Pediatrics (INP) is a major medical institution in Mexico, where patients with BMF receive a complete approach that includes paraclinical tests. Readily recognizable features, such as the hematological and distinctive physical phenotypes, identified by clinical dysmorphologists, remain crucial for the diagnosis and management of these patients, particularly in circumstances where next-generation sequencing (NGS) is not easily available. Here, we describe a group of Mexican patients with a high clinical suspicion of an IBMFS.

**Methods:** We performed a systematic retrospective analysis of the medical records of patients who had a high IBMFS suspicion at our institution from January 2018 to July 2021. An initial assessment included first ruling out acquired causes of BMF by the Hematology Department and referral of the patient to the Department of Human Genetics for physical examination to search for specific phenotypes suggesting an IBMFS. Patients with high suspicion of having an IBMFS were classified into two main groups: 1) *specific IBMFS*, including dyskeratosis congenita (DC), Diamond–Blackfan anemia (DBA), Shwachman–Diamond syndrome (SDS), thrombocytopenia with absent radii (TAR), and severe congenital neutropenia (SCN); 2) *undefined IBMFS (UI).*

**Results:** We established a high suspicion of having an IBMFS in 48 patients. At initial evaluation, the most common hematologic features were bicytopenia (20%) and aplastic anemia (16%); three patients received hematopoietic stem cell transplantation. Among patients with a suspicion of an IBMFS, the most common physical abnormality was minor craniofacial features in 83% of patients and neurodevelopmental disorders in 52%. The specific suspicions that we built were DBA (31%), SDS (18%), DC (14%), TAR (4%), and SCN (4%), whereas 27% of cases remained as undefined IBMFS. SDS, TAR, and SCN were more commonly suspected at an earlier age (<1 year), followed by DBA (2 years) and DC (5 years).

**Conclusions:** Thorough examination of reported clinical data allowed us to highly suspect a specific IBMFS in approximately 70% of patients; however, an important number of patients remained with suspicion of an undefined IBMFS. Implementation of NGS and telomere length measurement are forthcoming measures to improve IBMFS diagnosis in Mexico.

## Introduction

Bone marrow failure (BMF) is characterized by a decreased production of one or more hematopoietic lineages, which leads to diminished or absent hematopoietic precursors in the bone marrow, resulting in isolated cytopenias or pancytopenia in peripheral blood (Brodsky and Jones, 2005; Moore and Krishnan, 2023). According to its origin, BMF can be categorized into two groups, acquired or inherited (Fiesco-Roa et al., 2022). The distinction between acquired and inherited forms of BMF is crucial to offer adequate therapeutic interventions and appropriate genetic counseling.

Acquired BMF is often linked to immune-mediated events or exposure to environmental toxicity, chemotherapy, radiotherapy, or viral infections (Brodsky and Jones, 2005; Moore and Krishnan, 2023). To determine the underlying cause of BMF, a comprehensive assessment of each patient is necessary, including the record of environmental exposures, personal and family medical history, physical examination, and a complete laboratory work-up (Brodsky and Jones, 2005; Fassel and Sheth, 2020; Moore and Krishnan, 2023).

Inherited bone marrow failure syndromes (IBMFSs) are a group of rare disorders caused by germline pathogenic variants (GPVs) in genes associated with hematopoiesis and cellular maintenance (Dror, 2018). Collectively, the estimated incidence of the IBMFS is approximately 65 per million live births (Dror, 2018). The phenotypical presentation of the IBMFS is very heterogeneous. They frequently occur during infancy or childhood; however, an IBMFS can remain undiagnosed for years or may present cryptically in adult patients with an oligosymptomatic disease, often characterized by either unilineage or multilineage peripheral blood cytopenias (Alter, 2005).

Hematological abnormalities are the cardinal feature unifying the IBMFS, including an increased risk of developing BMF, myelodysplastic syndrome (MDS), acute myeloid leukemia (AML) and, more rarely, acute lymphoblastic leukemia (ALL), and other hematological neoplasias. However, they may also present with extra-hematological features such as dysmorphic features, congenital malformations, dysplasias, developmental delay, and short stature. In addition to the hematologic cancer risk, they also harbor an increased risk of solid tumors (Elghetany et al., 2021; Park, 2022; Deng and McReynolds, 2023). Even though a hematological overlap exists among the IBMFS, the extra-hematological characteristics can aid in distinguishing each other in a clinical setting (Alter, 2005).

The underlying causes of the different types of IBMFSs are diverse and affect several molecular pathways, including telomere shortening in dyskeratosis congenita (DC), deficient DNA interstrand crosslink repair in Fanconi anemia (FA), altered messenger RNA (mRNA) maturation in thrombocytopenia with absent radii (TAR), myeloid lineage growth arrest in severe congenital neutropenia (SCN), and defective ribosome biogenesis and processing, occurring in both Shwachman–Diamond syndrome (SDS) and Diamond–Blackfan anemia (DBA) (Elghetany et al., 2021; Park, 2022; Deng and McReynolds, 2023).

Diagnosis of IBMFSs is of utmost importance for genetic risk assessment and surveillance and the implementation of appropriate treatments, including reduced-intensity conditioning (RIC) regimens, treatment with androgens, and hematopoietic stem cell transplantation (HSCT), and for avoiding first-line treatment with immunosuppressive therapy (Fassel and Sheth, 2020; Deng and McReynolds, 2023).

The advent of genomic sequencing technologies using next-generation sequencing (NGS) has transformed the diagnostic approach of BMF. NGS has not only allowed precise and rapid turn-over diagnosis of suspected IBMFSs but also unveiled previously unrecognized Mendelian BMF syndromes with novel genetic etiologies (Bluteau et al., 2018). Unfortunately, high-throughput genomic sequencing technologies are not available in all clinical settings, particularly in low- and middle-income countries (LMICs). In Mexico, physicians mainly rely on clinical presentation for diagnosis. Therefore, expert dysmorphology training is a vital tool to detect and integrate an IBMFS diagnosis upon which management is planned.

Little is known about the prevalence and phenotypic spectrum of IBMFSs in the Mexican population, and available studies concentrate on FA (García-de Teresa et al., 2019; Reyes et al., 2022). To address current genotyping limitations, we implemented a clinical approach to expedite clinical suspicion and diagnosis. Here, we describe the clinical and hematologic characteristics of a group of Mexican patients with a high IBMFS suspicion, with an emphasis on the phenotypic spectrum. Although genotyping remains a short-term objective, our primary aim was to elucidate the clinical aspects of IBMFS within the Mexican population.

## Methods

### Data collection

We conducted a systematic retrospective analysis of the medical records of patients who attended the Medical Genetics outpatient clinic at our institution from January 2018 to July 2021 and included patients who met the criteria to suspect an IBMFS.

**FIGURE 1:**
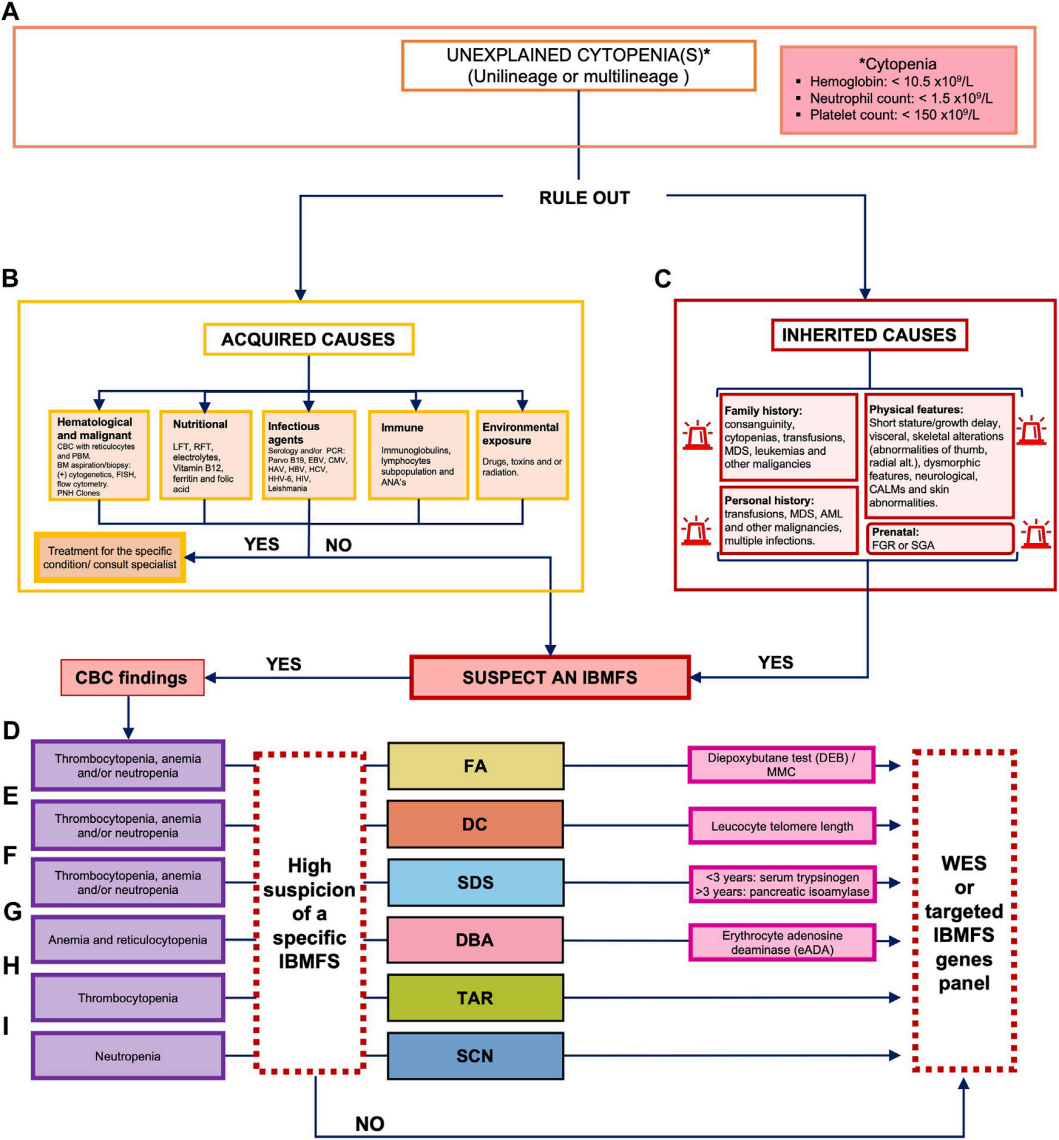
Decision algorithm for the clinical classification of patients with bone marrow failure. **(A)** Unexplained cytopenia(s) leading to the suspicion of an IBMFS can include a CBC of hemoglobin of <10.5 × 10^9^/L, neutrophils of <1.5 × 10^9^/L, and/or platelets of <150 × 10^9^/L. **(B)** The first decision point implies ruling out acquired causes of BMF including hematological malignancies and nutritional, infectious agents, immune, or drug exposure. **(C)** If acquired causes have been ruled out, we consider an IBMFS and complete a thorough physical examination, as well as prenatal, personal, and family history. Classification of the cytopenia and physical examination should be taken into consideration to guide clinical suspicion of a specific IBMFS. **(D)** If multilineage cytopenia and clinical features are compatible with FA, we perform the chromosome breakage test with DEB, if available. **(E)** If multilineage cytopenia and clinical features are compatible with DC, we perform the leukocyte telomere length test, if available (less often FA and DC can present with unilineage cytopenias). **(F)** If multilineage cytopenia and clinical features are compatible with SDS, we perform diagnostic screening with serum trypsinogen or pancreatic isoamylase. **(G)** If there is confirmation of anemia plus reticulocytopenia and clinical features are compatible with DBA, we perform diagnostic screening with erythrocyte adenosine deaminase (eADA). If a screening test is positive for a specific IBMFS, targeted testing should be considered for molecular confirmation. **(H)** If unilineage cytopenia (thrombocytopenia) and clinical features are compatible with TAR, genetic confirmation is followed. **(I)** If unilineage cytopenia (neutropenia) and clinical features are compatible with SCN, genetic confirmation is followed. In cases where a specific IBMFS suspicion is not made, but hereditary suspicion remains, we perform WES or a molecular panel, if available. ANAs, antinuclear antibodies; BMF, bone marrow failure; CALMs, cafe-au-lait macules; CBC, complete blood count; CMV, cytomegalovirus; DEB, diepoxybutane; EBV, Epstein–Barr virus; FA, Fanconi anemia; FGR, fetal growth restriction; HAV, hepatitis A virus; HBV, hepatitis B virus; HCV, hepatitis C virus; HHV-6, human herpesvirus type 6; HIV, human immunodeficiency virus; IBMFS, inherited bone marrow failure syndrome; LFT, liver function test; MDS, myelodysplastic syndrome; NGS, next-generation sequencing; PNH, paroxystic nocturnal hemoglobinuria; ParvoB19, parvovirus B19; PCR, polymerase chain reaction; RFT, renal function test; SGA, small for gestational age; PBS, peripheral blood smear; and WES, whole-exome sequencing.

Record review included a thorough examination of medical files/documents, laboratory results, and pathology and radiological reports. The collected information included demographics and pedigree data, results from imaging studies, and laboratory tests that confirmed the diagnosis of hematological abnormalities. It also included details from physical examination, details of factors such as multiple blood transfusions, hospitalizations due to infections, and malignancy, and information about therapeutic interventions, such as HSCT, chemotherapy, and administration of other drugs. We excluded patients with a positive diepoxybutane (DEB) chromosomal breakage test, which confirms a diagnosis of FA, as well as patients with potentially acquired causes, as determined by the Hematology Department through the pertinent studies (Figure 1A).

Using the diagnostic algorithm presented in the following section and based on our comprehensive analysis of the medical records, we suggested a potential IBMFS diagnosis. Since this study involved the review of medical charts, individualized participation consent forms were waived by the hospital’s Ethics and Research Committee. Informed consent for photograph use was obtained from the individuals whose pictures are presented in this study.

### Diagnostic algorithm

We reviewed medical records of patients referred by the Hematology Department who presented with an unexplained cytopenia, whether unilineage or multilineage. Anemia was determined by evaluating a patient’s hemoglobin (Hb) level in relation to age- and gender-specific normal ranges, typically with an approximate reference value of <10.5 g/dL. Neutropenia was defined by an absolute neutrophil count (ANC) of less than 1,500/μL and classified into two groups: severe neutropenia (<500/mm^3^) and non-severe neutropenia, which included mild (1,000–1,5000/mm^3^) and moderate neutropenia (500–1,000/mm^3^). Thrombocytopenia was diagnosed when the platelet count fell below 150,000/μL (Figure 1A).

Acquired causes were ruled out by analyzing specific laboratory work-up including at least a complete blood count (CBC) with reticulocytes, peripheral blood smear (PBS), liver and renal function tests, electrolytes, vitamin B12, ferritin, and folic acid. If any acquired cause was identified, targeted treatment was implemented. Other acquired causes have been linked to immune-mediated events or exposure to environmental toxicity such as history of chemotherapy, radiotherapy, or viral infections. To determine the underlying cause of BMF in our group of patients, we conducted a comprehensive assessment of the medical records. This assessment encompassed various factors, including environmental exposures, personal and family medical history, physical examination, and laboratory tests such as serology for ruling out viral infections and evaluation of immunoglobulins and lymphocyte subpopulations to assess potential immunodeficiencies and autoimmune diseases (Figure 1B).

The patient’s personal history was analyzed for “beacons,” suggesting a potential IBMFS. These beacons included 1) being born to consanguineous parents or belonging to an endogamic population or region (Bittles, 2001)—defined as having a population of less than 5,000 inhabitants (Del Castillo Ruíz et al., 2019))—or having a positive family history of cytopenias, transfusions, MDS, leukemia, and other hematological or solid malignancies that may or may not identify a clear inheritance pattern in the pedigree; 2) having a personal history of cytopenias, with early onset of MDS, leukemia, and other hematological or solid malignancies; 3) personal history of fetal growth restriction (FGR) or history of being born small for gestational age (SGA), and 4) the presence of physical alterations, including short stature, visceral and skeletal malformations, and/or dysmorphic features (Figure 1C).

We then evaluated CBC and reticulocyte values, looking for insights that could support the suspicion of a specific IBMFS.

**FIGURE 2:**
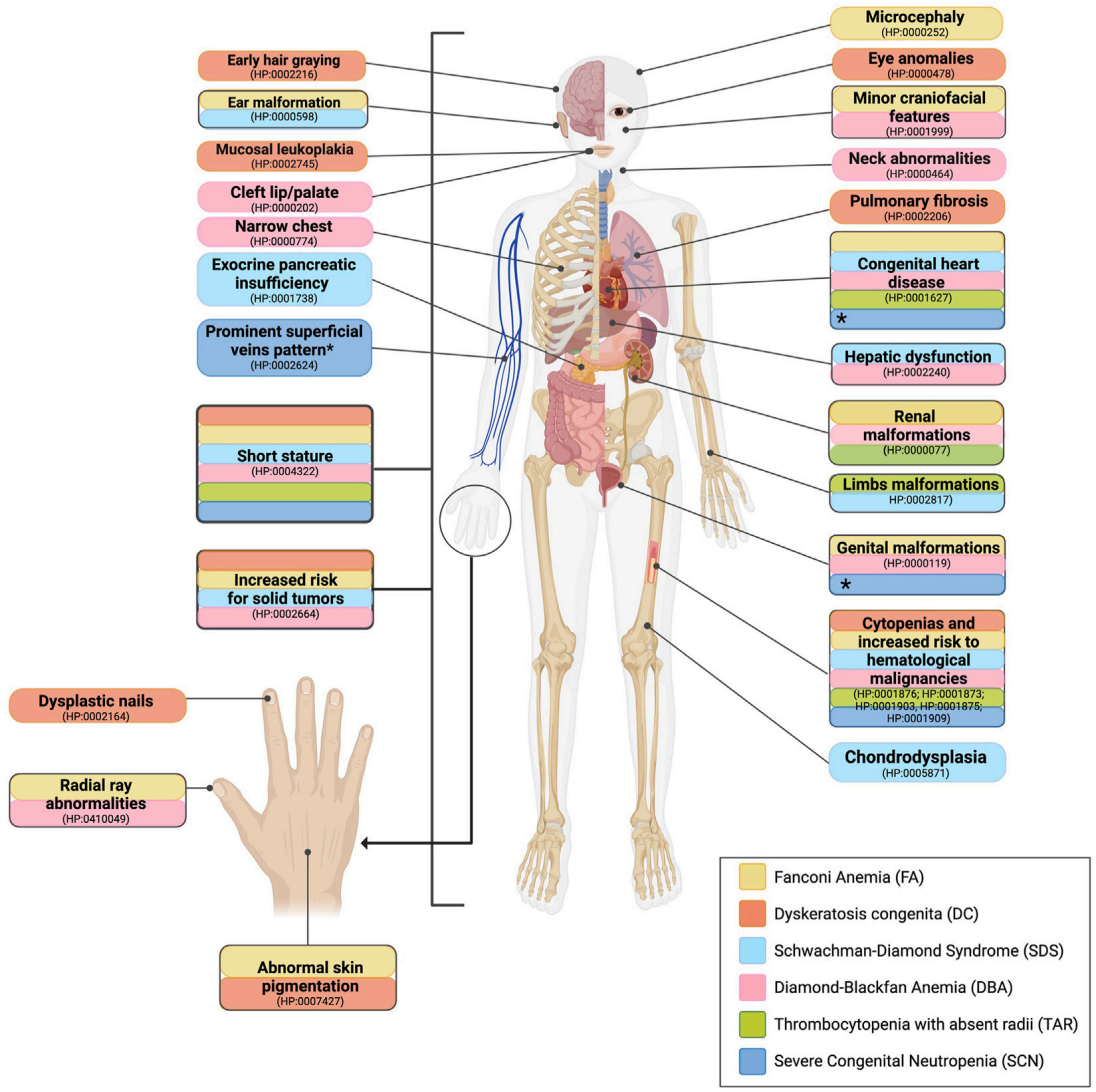
Comparative outline of the common anomalies observed in patients with IBMFS. Features that help build the suspicion of a specific IBMFS are given. The clinical manifestations of various IBMFS exhibit overlap in certain organs and systems. This includes cytopenias, an elevated risk of hematological malignancies, and short stature, which are common features among the classical IBMFS discussed in this paper. ***Highlighted in yellow***, patients with FA are characterized by short stature, abnormal skin pigmentation, facial features, congenital heart disease, ear malformations, genitourinary malformations, renal malformations, and thumb defects. ***Highlighted in orange***, patients with DC exhibit the following characteristics: nail dystrophy, mucosal leukoplakia, early hair graying, reticulated skin pigmentation, pulmonary fibrosis, short stature, and eye abnormalities. ***Highlighted in light blue***, patients with SDS present pancreatic insufficiency, skeletal dysplasia, short stature, congenital heart disease, and hepatic dysfunction. ***Highlighted in pink,*** patients with DBA present short stature, thumb defects, asymmetric scapula, fusion of cervical vertebrae, webbed neck, genitourinary malformations, and congenital heart disease. ***Highlighted in green***, patients with TAR present short stature, hypoplastic ulnae, hypoplastic humeri, and renal malformations. ***Highlighted in dark blue***, patients with SCN present with short stature and inflammatory bowel disease. *Increased visibility of superficial veins, congenital heart disease, and genitourinary malformations are commonly observed in patients with SCN harboring pathogenic variants in the *G6PC3* gene.

***FA*** was suspected in cases where the patient presented macrocytic anemia (HP:001972), thrombocytopenia (HP:0001873), and/or neutropenia (HP:0001875) in a CBC (Figure 1D). Suspicion increased when patients had VACTERL-H (MIM#192350) or PHENOS features, defined as abnormalities in pigmentation (such as abnormal skin), head (microcephaly), and eyes (including short palpebral fissures and microphthalmia), neurological manifestations, otology-related issues, and short stature even without hematological alterations (Figure 2, yellow boxes) (Fiesco-Roa et al., 2019). The gold standard for confirming FA suspicion is a positive DEB test (Molina et al., 2022).

***DC*** was suspected if the patient presented with anemia (HP:001,972), thrombocytopenia (HP:0001873), and/or neutropenia (HP:0001875) in a CBC (Figure 1E) and if the patient exhibited at least one manifestation of the mucocutaneous triad of oral leukoplakia (HP:0002745), dysplastic nails (HP:0002164), and reticulated skin pigmentation (HP:0007427). Other clinical manifestations such as early hair graying (HP:0002216), short stature (HP:0004322), eye anomalies (HP:0000478), and pulmonary fibrosis (HP:0002206) were also considered as they have been observed in patients with DC, aiding in the diagnosis (Figure 2, orange boxes). Leukocyte telomere length (LTL) serves as a functional diagnostic screening test with high sensitivity for DC when available. It is considered a gold standard after genotyping genes linked to the DC phenotype or whole-exome sequencing (WES). In cases of high DC suspicion, we currently order LTL tests through external facilities. However, as we are in the process of establishing an in-house LTL test through RT–PCR and Flow-FISH, these tests will soon be accessible for Mexican patients strongly suspected of having DC.

***SDS*** was suspected if a patient exhibited anemia (HP:001972), thrombocytopenia (HP:0001873), and/or neutropenia (HP:0001875) in a CBC (Figure 1F). Pancreatic insufficiency (PI) was considered when a 72-h fecal fat qualitative test (FFQT) was positive. Clinical suspicion of SDS increased when the patient presented with at least three of the following associated features: exocrine pancreatic dysfunction (EPI) (HP:0001738), malabsorption syndrome (HP:0002024), short stature (HP:0004322), chondrodysplasia (HP:0005871), thoracic abnormality (HP:0045027), congenital heart defect (HP:0001627), otological malformations (HP:0000598), and hepatomegaly (HP:0002240) (Figure 2, light blue boxes).

***DBA*** was suspected if the CBC reports macrocytic anemia (HP:001972) along with reticulocytopenia (HP:0001896) (Figure 1G). HbF is a parameter often found elevated in DBA, although not unique. Considered minor features were short stature (HP:0004322), microcephaly (HP:0000252), minor craniofacial features (HP:0001999), neck abnormalities (HP:0000464), upper limb malformations (HP:0002817), congenital heart defect (HP:0001627), and genitourinary malformations (HP:0000119) (Figure 2, pink boxes).

***TAR*** was suspected in patients exhibiting both thrombocytopenia (HP:0001873) (Figure 1H) and skeletal abnormalities (HP:0000924) that showed minor-to-major alterations in radial ray (HP:0410049). Unlike FA, TAR patients preserve the thumbs. Associated features such as congenital heart defect (HP:0001627) and renal alterations (HP:0000077) further contributed to the suspicion (Figure 2, green boxes).

***SCN*** was suspected when patients had neutropenia, ≤1,5000/mm^3^ (HP:0001875) throughout their lifetime (Figure 1I) and two or more associated features including short stature (HP:0004322), congenital heart defect (HP:0001627), and urogenital alteration (HP:0000119) (Figure 2, dark blue boxes).

Patients with a well-supported suspicion of having an IBMFS but in whom a specific clinical diagnosis was not possible were categorized as having an undefined IBMFS.

### Inclusion criteria

We included patients with clinical suspicion of an IBMFS who had anemia, neutropenia and/or thrombocytopenia, bicytopenia, BMF, and/or the presence of two or more non-hematological manifestations associated to specific IBMFS. Likewise, to consider an IBMFS, the patient had to meet at least one of the following criteria (Supplementary Figure S1):

• Positive family history (consanguinity/endogamy and/or family history of hematological disorder)
• Physical abnormality
• Initial hematologic alteration at <18 years of age

### Exclusion criteria

We did not include patients with a likely acquired BMF and acquired aplastic anemia (HP:0001915) or cases of paroxysmal nocturnal hemoglobinuria (PNH) (HP:0004818). Patients with a positive chromosomal breakage test with DEB in peripheral blood were excluded from this study since a confirmatory FA diagnosis was reached (Supplementary Figure S1).

### Statistical analysis

Collected data were entered and analyzed using GraphPad Prism 10 Statistics Guide (United States). For categorical variables, we used chi-square (χ2), while analysis of variance (ANOVA) was used for continuous variables. Quantitative (numerical) variables are presented as mean, standard deviation, median, maximum, and minimum, while qualitative (categorical) variables were summarized as frequencies and percentages.

## Results

### Demographics of patients with a suspected IBMFS

**FIGURE 3:**
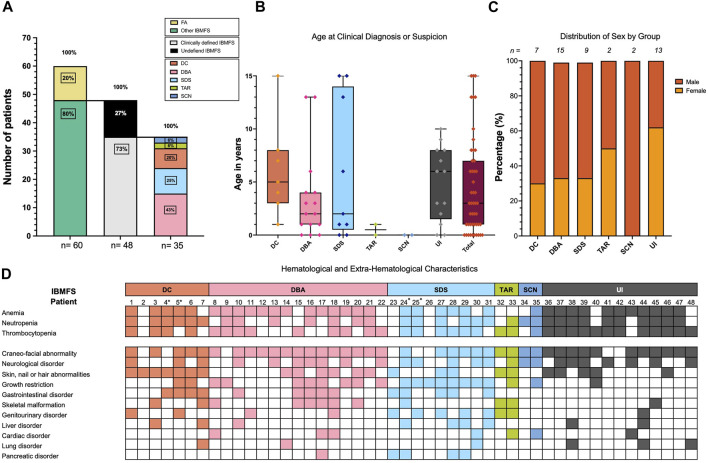
Demographics and clinical characteristics of 48 patients with high suspicion of having an IBMFS. **(A)** A total of 60 patients were suspected to have an IBMFS. In 12 (20%) patients, a positive chromosome breakage DEB test confirmed FA. A total of 48 (80%) patients remained with a strong suspicion of having an IBMFS. By following the clinical criteria outlined in Figures 1, 2, we were able to build a high suspicion of a specific IBMFS in 35 (73%) of these patients. Despite having a strong notion of a genetic condition, we were unable to assign a specific IBMFS in 13 (27%) patients and they remained as having an undefined IBMFS. Frequencies of the specific IBMFS suspected in 35 patients are indicated in the rightmost stacked bar plot. **(B)** Boxplot showing the age at clinical diagnosis or suspicion of a specific IBMFS across patients. **(C)** Distribution of males and females per group. **(D)** Hematological and extra-hematological characteristics of 48 patients with high suspicion of having an IBMFS. *Patients 4 and 5 are siblings in the DC group, and patients 24 and 25 are siblings in the SDS group.

We identified a total of 60 patients with high suspicion of having an IBMFS which met the inclusion criteria. We evaluated their medical records using our diagnostic algorithm (Figures 1–3A). Twelve patients had a positive DEB test confirming FA and were excluded from further evaluations. A total of 48 patients from 46 unrelated families with high suspicion of having an IBMFS were included in this study. Sex distribution was 1:1.3 female-to-male ratio. The mean age at the time of medical record evaluation was 9.02 years, with most patients presenting hematological alterations that began during their pediatric years (Table 1).

**TABLE 1:** General characteristics of 48 patients with high suspicion of having an IBMFS.

Characteristic	Total (*n* = 48)
Sex	Number (%)
Male	29 (60)
Female	19 (40)
Mean age (years)	9.02 (±6.01)
Age at hematological onset	Number (%)
≤ 2	22 (49)
> 2 and ≤ 5	9 (20)
> 5 and < 18	14 (31)
Family history	Number (%)
Endogamy	11 (23)
Consanguinity	4 (8)
Isonymy	3 (6)
Family history of hematological disorders	2(4)

The following information is described: male:female ratio, age at onset of hematological alterations, and family history data.

Eight percent (4/48) of the patients were born from consanguineous marriages, with a degree of consanguinity of the third or fourth degree. A notable portion of our group of patients 23% (11/48) originated from endogamic regions, primarily concentrated in the central regions of Mexico. One of such families also shared a surname. Only two patients had a documented family history of hematological and/or IBMFS disorders; their phenotypes corresponded to the DC and SDS groups, as shown in Figure 3D with an asterisk (*), and detailed pedigrees are provided in Supplementary Figure S2.

Out of the 48 patients, 73% (35/48) exhibited overt symptoms suggestive of an IBMFS. Following our diagnostic algorithm, the medical record analysis allowed us to classify them as probable DC in 20% (7/35), probable SDS in 25% (9/35), probable DBA in 43% (15/35), probable TAR in 6% (2/35), and probable SCN in 6% of patients (2/35). The remaining 27% (13/48) were left with the diagnosis of a probable undefined IBMFS (Figure 3A).

In total, 4/48 (8%) patients died. Two fatalities in the DC group were due to pulmonary infections at 18 and 12 years of age. Two other mortalities occurred in the UI group, resulting from an infection and primary severe AA complications at the ages of 7 and 12 years, respectively.

There was no statistically significant difference in the age at which probable IBMFS diagnoses were made across the various IBMFS subtypes. Overall, the median age for suspicion of IBMFS was 3 years. Specifically, the median ages for the UI, DC, DBA, and SDS groups were 6, 5, 2, and 2 years, respectively, and for TAR and SCN, the median age was less than 1 year (Figure 3B). Sex distribution had no statistically significant differences between groups (Figure 3C).

### Physical phenotype of patients with a suspected IBMFS

In addition to hematological features, the physical characteristics of the 48 patients are summarized in Figure 3D. Craniofacial abnormalities (HP:0001999) were the most reported feature and were present in 40 out of 48 individuals (83%). Approximately half of the patients (52%) (25 out of 48) exhibited neurological abnormalities (HP:0000707), including structural central nervous system (CNS) abnormalities (HP:0002011), developmental delay (HP:0012758), intellectual disability (HP:0001263), cognitive impairments (HP:0100543), and seizures (HP:0001250).

Skin, nail, and/or hair abnormalities (HP:0001574) were present in 48% (23/48) of patients. Among skin changes, hyper/hypopigmented macules (HP:0007441) and café-au-lait macules (CALM) (HP:0000957) were documented. Detailed characteristics of each patient are depicted in Supplementary Table S1.

Growth restriction (HP:0001510) was documented in 44% (21/48) of the patients; however, certain groups have a higher frequency of growth restriction: 89% (8/9) for SDS and 53% (8/15) for DBA (Figure 3D).

Gastrointestinal disorders (HP:0011024) were found in 29% (14/48) of the patients, followed by skeletal anomalies in 25% (12/48) and genitourinary alterations (HP:0000119) in 25% (12/48). Additionally, liver (HP:0001392), cardiac (HP:0001627), lung (HP:0002088), and pancreatic disorders (HP:0001732) were present in 19%, 14%, 12%, and 10% of the cases, respectively (Figure 3D; Supplementary Table S1).

As expected, in the group of patients with high suspicion of DC, the integument features were the most common; at least one feature of the mucocutaneous triad was present in all patients. Individual components of the triad were observed in at least half of the patients: leukoplakia (HP:0002745) in 57% (4/7), nail alterations (HP:0001597) in 71% (5/7), and hyper/hypopigmented skin alterations (HP:0007441) in 100%. The complete triad was only found in 57% (4/7) of these patients.

In patients with high suspicion of having SDS, the combination of EPI (HP:0001738) and neutropenia (HP:0001875) was found in 44% (4/9). Malabsorption syndrome (MS) (HP:0002024) dominated in 55% (5 patients), suggesting EPI, but the FFQT was not performed in all the patients. Remarkably, neurological alterations were present in 55% (5/9) of patients in this group.

The physical presentation of the group with high DBA suspicion was one of the most heterogeneous, and the medical records documented at least one minor craniofacial feature (HP:0001999).

The two patients in the group of highly probable TAR presented different phenotypic alterations of the radial ray. These ranged from type II radial longitudinal deficiency (RLD) and unilateral radius agenesis to bilateral radius agenesis type IV RLD (Bayne and Klug, 1987). Both patients had preservation of the thumbs, although the thumb was hypoplastic in the patient with a unilateral absent radius (HP:0009601). In addition, neurological alterations, specifically moderate developmental delay, were reported in one of the patients from this group.

The two patients in the SCN group presented neurological alterations. One of them also presented associated anomalies such as congenital heart defect (HP:0001627), characterized as a valvulopathy, affecting the mitral valve moderately alongside mild tricuspid and aortic insufficiency, short stature (HP:0004322), and developmental delay (HP:0012758).

Three patients exhibited clinical features that strongly suggested an IBMFS despite lacking a history of hematological abnormalities. Specifically, patient 2 had the clinical mucocutaneous triad highly suggestive of DC. Patients 23 and 26 presented EPI (HP:0001738), a hallmark of SDS. Patients 42 and 43 had no apparent physical alterations, yet their early diagnoses of MDS (HP:0002863) and AA (HP:0001915) at 8 and 7 years of age, following the exclusion of acquired causes, strongly raise the suspicion of an underlying IBMFS (Figure 3D).

### Phenotype of selected illustrative cases

In this section, we illustrate three cases with a high suspicion of an IBMFS diagnosis, specifically DBA, SDS and DC, and how the clinical suspicion was adjusted after using our diagnostic algorithm. The benefit of genotype confirmation is also exemplified, as this was possible for one of these cases.

#### Confirmed case of DBA

**FIGURE 4:**
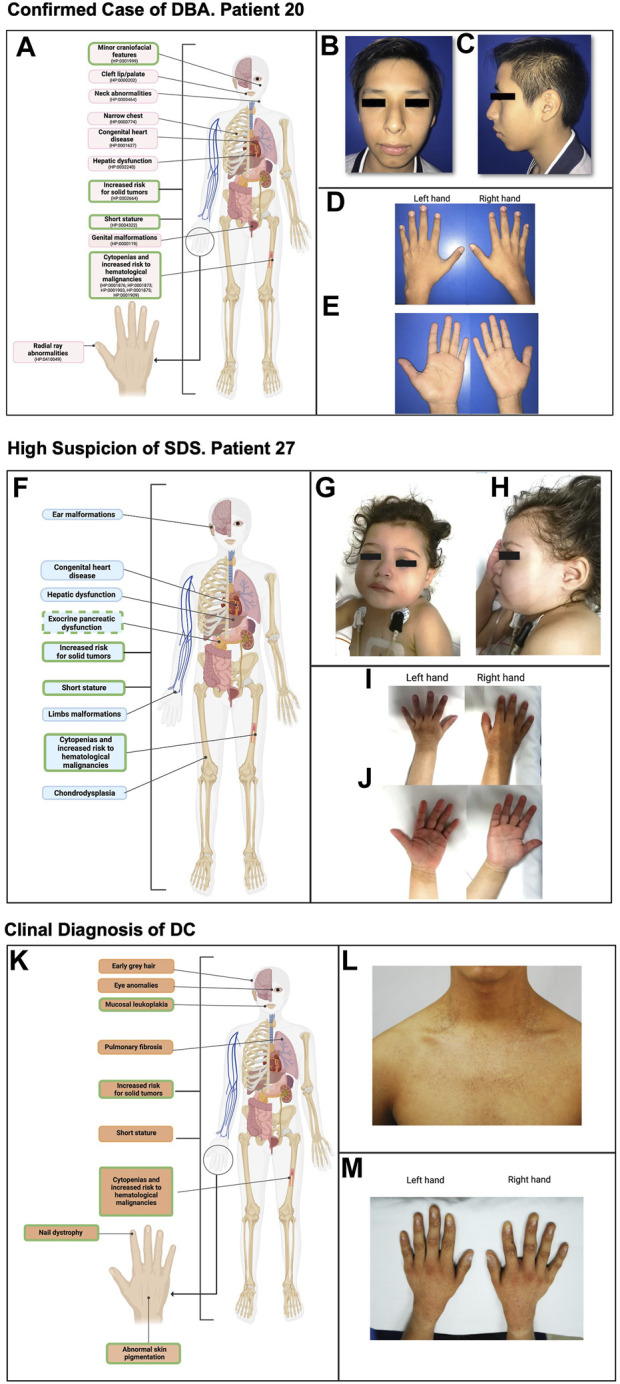
Phenotype of selected cases with high suspicion of having an IBMFS. **(A)** Highlighted in pink are the typical phenotypic anomalies observed in patients with DBA. Outlined in green are the clinical manifestations found in this patient. **(B)** Frontal facial photography showing craniofacial features, including an elongated face and bulbous nasal tip. **(C)** Lateral facial photography showing craniofacial features, including midface hypoplasia and apparent micro-retrognathia. **(D, E)** Left and right hands in both supine and prone positions where no abnormalities were found. **(F)** Highlighted in light blue are the typical phenotypic anomalies observed in patients with SDS. Outlined in green are the clinical manifestations found in this patient. Exocrine pancreatic dysfunction is outlined with a dashed line since this patient presents with malabsorptive syndrome; however, exocrine pancreatic dysfunction laboratory work-up remains pending. **(G)** Frontal facial photography showing craniofacial features, including epicanthal folds. **(H)** Lateral facial photography showing craniofacial features, including a prominent antihelix. **(I, J)** Left and right hands in both supine and prone positions where no abnormalities were found. **(K)** Highlighted in light orange are the typical phenotypic anomalies observed in patients with DC. Outlined in green are the clinical manifestations shared by the patient. **(L)** Frontal neck and upper part of the thorax photography showing hyper/hyporeticular pigmentation of the skin. **(M)** Left and right hand in prone position where dysplastic nails are observed in all 10 fingers.

Patient 20 had a strong IBMFS suspicion, due to early onset of hematological manifestations at 8 months, featuring macrocytic anemia and reticulocytopenia, followed by the development of severe neutropenia at the age of 4 years. Upon physical examination, subtle craniofacial features, including an elongated face (HP:0000276), bulbous nasal tip (HP:0000414), midface hypoplasia (HP:0011800), and apparent micro-retrognathia (HP:0000308), were recorded (Figures 4A–E). Running this case through the proposed algorithm suggested a potential diagnosis of DBA. The suspicion was later confirmed by the NGS panel (Bone Marrow Failure Syndrome Panel; Illumina, San Diego CA, United States), detecting a heterozygous monoallelic pathogenic variant in *RPS19* [NM_001022.3: c.185G>C, (p.Arg62Pro)], with no report in population databases, previously reported and associated to DBA. This case exemplifies the potential oversight of minor craniofacial features in addition to associated hematological alterations with or without major malformations. Systematic analysis of facial characteristics in DBA may expand the phenotypic spectrum of DBA to include them as part of the clinical presentation and may consider them as major criteria for clinical diagnosis.

#### High suspicion of SDS

Patient 27 had an IBMFS suspicion due to severe neutropenia, associated with recurrent infectious episodes requiring hospitalization. Her extramedullary features included a malabsorptive syndrome (HP:0002024) and a genitourinary disorder (HP:0000119), attributed to nephrolithiasis (HP:0000787), along with short stature (HP:0004322) and bilateral epicanthal folds (HP:0000286) (patient 27, Figures 4F–J). Our algorithm straightforwardly classified this patient as having a probable SDS; the suspicion was later supported by her clinical progression, as the patient went on to develop both anemia and thrombocytopenia showing a classic course for SDS.

#### Clinical diagnosis of DC

The subsequent patient has been included in order to exemplify a DC case within the Mexican population, although he is not part of the DC group analyzed in this report.

He is a 17-year-old patient, (Figures 4K–M), who had an IBMFS suspicion due to severe thrombocytopenia at the age of 7. Three years later, he developed mild anemia without the need of blood transfusions. Due to persistent thrombocytopenia, a BM biopsy was performed at 14 years of age, showing hypocellularity consistent with BM aplasia. Clinical assessment and patient history uncovered nail dystrophy documented since the 7 years of age, initially misdiagnosed as lichen planus. Further evaluation revealed oral leukoplakia and hyperpigmented skin lesions as the extramedullary features (Figure 4K). Subsequently, clinical evaluation reinforced the suspicion of DC, as the patient exhibited the classic triad of dysplastic nails, reticular pigmentation of the neck, and oral leukoplakia, consistent with this condition (Figures 4L–M). This case underscores the importance of thorough clinical evaluation for a timely diagnosis and the subsequent implementation of appropriate treatment for the complex multisystemic conditions associated with this disorder.

### Hematological outcomes

Hematologic alterations were a prevalent finding, with 94% (45/48) of patients developing such alterations in different pediatric stages. Notably, 49% (22/45) had onset before or at 2 years of age, 20% (9/45) between 3 and 5 years, and the remaining 31% (14/45) between 6 and 17 years (Table 1).

**FIGURE 5:**
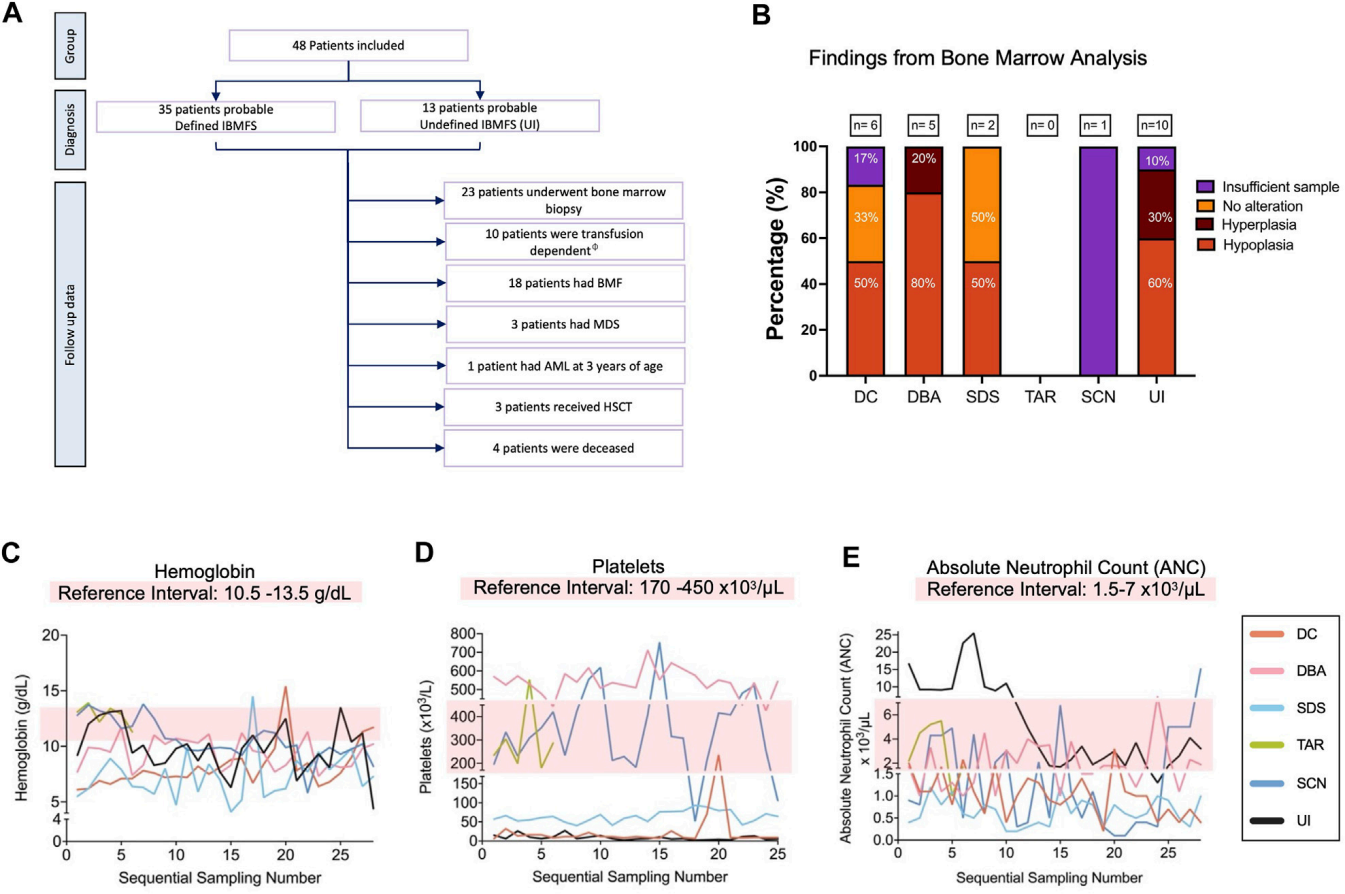
Hematological findings in 48 patients with high suspicion of having an IBMFS. **(A)** Overview of the hematologic follow-up in the 48 patients with high suspicion of having an IBMFS. **(B)** Bone marrow analysis. Bar graph shows the number of patients per group that underwent a BM biopsy/aspirate and their pathological findings. **(C)** Hemoglobin (Hb) levels over time. The light pink box indicates the normal reference interval, counts below this level indicate anemia. One representative patient per group is shown. **(D)** Platelets over time. The light pink box indicates the normal reference interval, counts below this level indicate thrombocytopenia. One representative patient per group is shown. **(E)** ANC over time. The light pink box indicates the normal reference interval, counts below this level indicate neutropenia. One representative patient per group is shown. ^ϕ^Transfusion dependency is defined as patients requiring ≥2 units of RBCs per 28 days (Germing et al., 2019).

Twenty-one percent (10/48) were transfusion-dependent patients. Among the 23 patients who underwent a bone marrow biopsy, hypocellular bone marrow (HP:0005528) was identified in 33% (16/48) (Figures 5A, B). Three patients developed MDS (HP:0002863), and one patient progressed to AML (HP:0004808). Three patients received hematopoietic stem cell transplantation (HSCT): a haploidentical transplant in a patient with DBA suspicion and two allogenic transplants, one patient from the DBA group and the other from the UI group. The latter patient went on to develop a low-grade mucoepidermoid carcinoma, with a lower lobe intrabronchial location at the age of 16 years (Supplementary Table S1; Figure 5A).

Most patients had recurrent CBC sampling. Approximately half of the patients (48%) (23/48) developed pancytopenia (HP:0001876). The remaining patients exhibited cytopenias in one or two lineages, with the presentation varying. In most cases, this cytopenia presented as either isolated anemia or thrombocytopenia. However, patients who presented with bicytopenia had both anemia and either neutropenia or thrombocytopenia.

Longitudinal CBC data from selected patients showed hemoglobin (Hb) fluctuations, with most of the patients having Hb reductions over time (Figure 5C). Persistent severe thrombocytopenia (HP:0001873) was observed in patients with high suspicion of DC, SDS, and UI. The patient with SCN suspicion showed thrombocytopenia in two separate occasions, which has been observed in different types of SCN, including SCN4 (MIM#612541), although an infectious episode cannot be discarded. The patients with TAR and DBA suspicions presented highly fluctuating platelet levels (Figure 5D). Leukopenia (HP:0001882) was observed in all patients, except for the TAR-suspected patient. All patients consistently displayed neutropenia (HP:0001875). The patient from the SCN group (patient 35) presented the lowest ANC value, falling below 200 x cells/μL, closely followed by the patient with SDS (patient 24) (Figure 5E).

Hematological abnormalities within the SDS suspicion group were observed with an average age of onset at 2 years, with three patients experiencing hematological alterations between 6 and 12 months. In our SDS suspicion group, neutropenia was identified in 66.6% (9/6 patients), with severe neutropenia (HP:0001875) in 44.4% (4/9) and non-severe neutropenia in 22.2% (2/9). The remaining 33.3% (3/9) did not have neutropenia but presented other clinical features suggestive of SDS.

Forty percent (6/15) of patients with DBA suspicion experienced hematological alterations (HP:0001871) within the first year of life, and 80% (12/15) presented with hematological alterations before 5 years of age. In the DBA suspicion group, 33% (5/15) of patients underwent BM studies. Furthermore, 80% (4/5) of patients had BM hypoplasia (HP:0005528), specifically of the myeloid–erythroid cell lineage. The two patients in the SCN suspicion group exhibited cyclic neutropenia (HP:0040289) documented in their CBC.

## Discussion

Published information on cohorts of patients with IBMFS from LMICs, including Mexico, remains scarce. Comprehensive available data of Mexican patients with IBMFS are predominantly case reports or small case series, such as those for patients with FA (García-de Teresa et al., 2019; Reyes et al., 2022), or case reports from a limited number of patients with DC or SCN (López-Rodríguez et al., 2022; Picos-Cárdenas et al., 2022; Velez-Tirado et al., 2022).

In high-income countries, the advent of panel-based NGS strategies has shifted the diagnosis of the IBMFS toward a “genotyping first” approach (Wetterstrand, 2023). However, in LMICs, the communion between laboratory tests and phenotypic examination remains the Rosetta stone for guiding the clinicians to use confirming molecular genotyping tests. The latter is particularly true in contexts where the patients cannot afford big expenses. Therefore, in Mexico, the school of human geneticists with profound dysmorphology training is king, guaranteeing the precise documentation of even subtle clinical features and guiding clinical follow-up of patients while waiting for genotype confirmation.

In this study, we describe a group of 48 patients followed up at our hospital in Mexico. These patients presented cardinal hematological alterations and a dysmorphological phenotype leading us to a high level of suspicion for them to have an IBMFS. In 35 of these patients, phenotype and laboratory tests made us build the suspicion of a specific IBMFS, including DC, DBA, SDS, TAR, and SCN. In the remaining 13 patients, acquired causes were ruled out, and multiorgan involvement was present; however, even after the application of our algorithm, we did not collect sufficient information to assign these patients to a specific disease.

Already existing clinically validated diagnostic criteria for DC and DBA were applied (Dokal, 2000; Vlachos et al., 2008; Bertuch, 2022; Niewisch et al., 2022) and made the suspicion of these two IBMFSs a more straightforward task. For identifying patients with DBA, the validated criteria focus on hematological data and age of presentation, considering a clinical diagnosis with four major criteria. Only one patient met the criteria for being a classic DBA, including being <1 year old, anemia with co-occurrent reticulocytopenia, and paucity of erythroid precursors in the BM. The remaining patients have what may be a non-classical DBA and met the supporting criteria of the presence of congenital anomalies associated with classical DBA. For identifying patients with DC, we searched for the validated mucocutaneous triad in patients with BMF. The complete triad was present in four out of the seven (57%) patients, which is consistent with the 30%–46% reported in the literature (Shimamura and Alter, 2010; Ward et al., 2018; Niewisch et al., 2022). At least one characteristic of the triad was present in all these seven patients (7/7).

Validated criteria for identifying patients with suspicion of SDS, TAR, and SCN have not been published. This may be attributed to the absence of established associations or the complexities arising from their broad phenotypic spectrum. It is important to note that rare diseases, such as the IBMFS, face significant research challenges due to limited patient numbers. SDS diagnosis is particularly challenging in our region due to the scarcity of tests for measuring isoamylase and trypsinogen and to perform the FFQT. However, we suspected SDS when the patient had hematological findings, dysmorphological features, and a history of pancreatitis or a positive FFQT. Therefore, we emphasize the critical need for periodic laboratory monitoring for the diagnosis of pancreatic insufficiency in this patient group, as enzyme replacement therapy can effectively manage the associated symptoms (Nelson and Myers, 2018).

To identify potential cases of SCN, we accounted for pivotal features, such as recurrent infections, short stature, congenital heart defects, and craniofacial features. Regarding cases with TAR suspicion, we focused on radial ray alterations, ranging from hypoplasia to agenesis, with or without congenital heart or renal defects, along with a history of thrombocytopenia.

Broadly speaking, most of our patients presented at least one minor craniofacial abnormality (85%), including palpebral fissures (up-slant or down-slant), epicanthal fold, anteverted nares, retrognathia, and/or frontal bossing. Craniofacial abnormalities were most of the time accompanied by a neurological disorder, increasing the suspicion of a genetic/syndromic etiology (Decimi and Memo, 2015). Short stature was also a prevalent feature (44%). The frequency of short stature in our group was 53% for DBA suspicion, 88% for SDS suspicion, and 28% for DC suspicion, consistent with previous reports for DBA (30%–38%) and SDS (69%) (Chen et al., 2005; Thompson et al., 2022; Wan et al., 2022). A clear picture of short stature prevalence was not established in the remaining groups due to the small number of patients.

Incidence of SCN and TAR was less common, consistent with the low prevalence reported worldwide (Martínez-Frías et al., 1998; Zeidler et al., 2009; Donadieu et al., 2013). The diagnosis of SCN syndrome is challenging, given that neutropenia is often cyclic (Donadieu et al., 2013). Of the two patients, number 34 (Figure 3D) presented only DD, which could be part of the non-syndromic *G6PC3* cases or Kostman’s syndrome, which is mainly associated with neurological alterations. Finally, prenatal or at birth detection of unilateral or bilateral radial anomalies should prompt the search for thrombocytopenia. In a cohort of 26 patients with bilateral radial agenesis, 73% presented with thrombocytopenia (Boussion et al., 2020). Therefore, the detection of radial ray anomaly requires the inspection of fluctuating thrombocytopenia or genetic molecular testing to confirm the suspected condition or differential diagnoses.

Although FA patients were excluded from this study, it is important to note that the FA phenotype may share similarities in physical and hematological characteristics with specific IBMFS, such as DBA and TAR. These similarities include alterations in the extremities, specifically in the radial ray, as well as congenital heart disease, genitourinary malformations, and renal malformations. When there is clinical suspicion of FA, a comprehensive evaluation and specialized testing such as the DEB chromosomal breakage test in skin fibroblasts when appropriate to rule out mosaic forms of FA and/or NGS testing are essential.

An important limitation of our study is the scarcity of molecular confirmatory testing which was conducted in only one patient (1 out of 48). Genotyping is a desired step in the study of IBMFS, as it not only allows confirming the clinical diagnosis but also results in comprehensive and accurate genetic counseling; it may aid in identifying other at-risk relatives and in determining whether the condition is *de novo* or inherited from a carrier or an affected parent with a cryptic presentation. It is noteworthy that in this study, the patients’ progenitors self-reported no underlying medical conditions, but without genotype analysis, incomplete penetrance of certain conditions cannot be ruled out (Fassel and Sheth, 2020; Elghetany et al., 2021).

Finally, while providing a timeframe for diagnostic opportunity would be desirable, in LMICs, providing an explicit diagnostic timeframe is challenging for multiple reasons, including, but not limited to, the fact that the diagnostic process for a patient presenting an IBMFS is highly individualized, influenced by factors such as case complexity, regional healthcare infrastructure, and resource availability. Additional variables include the choice of a diagnostic laboratory inside or outside the reach of the healthcare provider of the patient and the local availability of genetic testing. Furthermore, the selected genetic tests can significantly affect the diagnostic timeline. In countries where budget is not a limitation, or in cases where patients pay for genetic testing out of pocket, the diagnostic process typically takes between 3 and 8 weeks, depending on the laboratory and genetic test, i.e., panel sequencing or exome sequencing. Therefore, we stress the key relevance of close work with the hematology team.

In the near future, we will launch a national registry of patients with IBMFS, which will include the genotyping of all patients through WES. Identifying the genotype of these patients will test the performance of the clinical diagnostic algorithm presented in this study within the Mexican population affected by IBMFS.

## Conclusions

In this study, we describe phenotypes that, when correlated with cardinal hematological findings, could serve as indicators for high suspicion and the clinical diagnosis of different IBMFSs. Likewise, we describe specific clinical characteristics that should raise suspicion of DC, DBA, SDS, TAR, and SCN. This, in turn, could enhance the feasibility of personalized follow-up and management strategies, considering the need for adjusted dosing regimens in IBMFS cases, until molecular confirmation is attainable.

## Data Availability

The original contributions presented in the study are included in the article/Supplementary Material; further inquiries can be directed to the corresponding author.

## Supplementary material

SUPPLEMENTARY TABLE S1 Detailed characteristics of 48 patients with high suspicion of having an IBMFS.

SUPPLEMENTARY FIGURE S1 Patients with a high suspicion of having an IBMFS at our medical institution. Patients affiliated with the Instituto Nacional de Pediatria who attended the institution from January 2018 to July 2021 were considered. Criteria for the inclusion and non-inclusion of patients with IBMFS are detailed. The included patients presented with a classical phenotype of an IBMFS, central-origin cytopenias, AML or MDS in the pediatric years, associated with a family history of BMF or cancer, and/or a physical abnormality. We excluded patients with likely acquired BMF as well as those who had a confirmed diagnosis of FA from this analysis. A total of 48 patients were included. *FA was discarded in all patients with the DEB test in peripheral blood. Patients who tested positive for DEB were excluded from this study due to their inclusion in another cohort specific for FA.

SUPPLEMENTARY FIGURE S2 Pedigree of two unrelated families. **(A)** Pedigree of patients 4 and 5 (II1 and II2, respectively) with clinical suspicion of DC. **(B)** Pedigree of patients 24 and 25 (II1 and II2, respectively) with clinical suspicion of SDS. Affected individuals are shaded in black.
