# Supplementary figures and images for: Inherited bone marrow failure syndromes: phenotype as a tool for early diagnostic suspicion at a major reference center in Mexico
Source: Front Genet. 2024 Jan 24;14:1293929. doi: 10.3389/fgene.2023.1293929 (PMC10848162; doi:10.3389/fgene.2023.1293929)

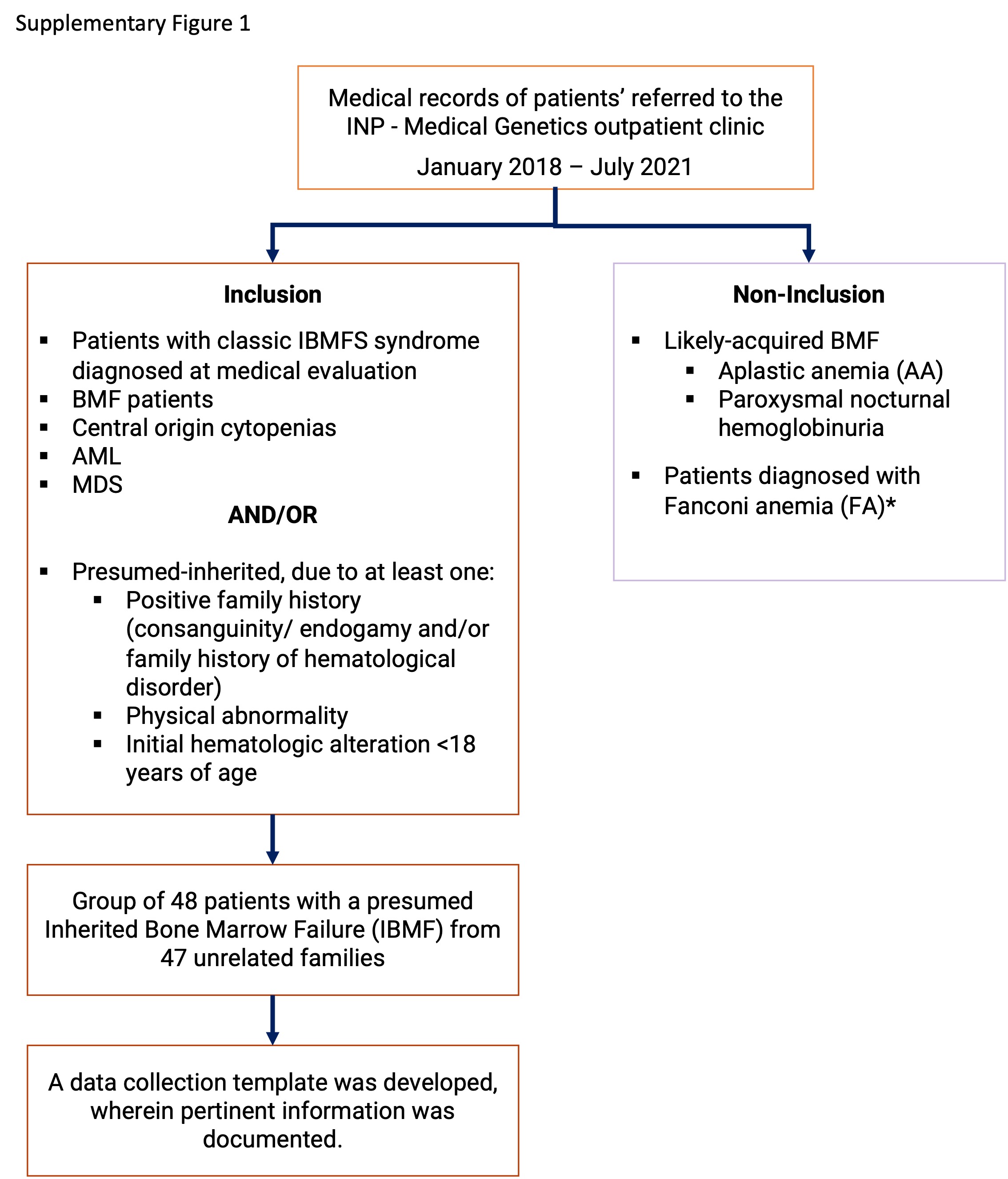

Supplement: Supplementary file 1 [file Image1.jpeg]

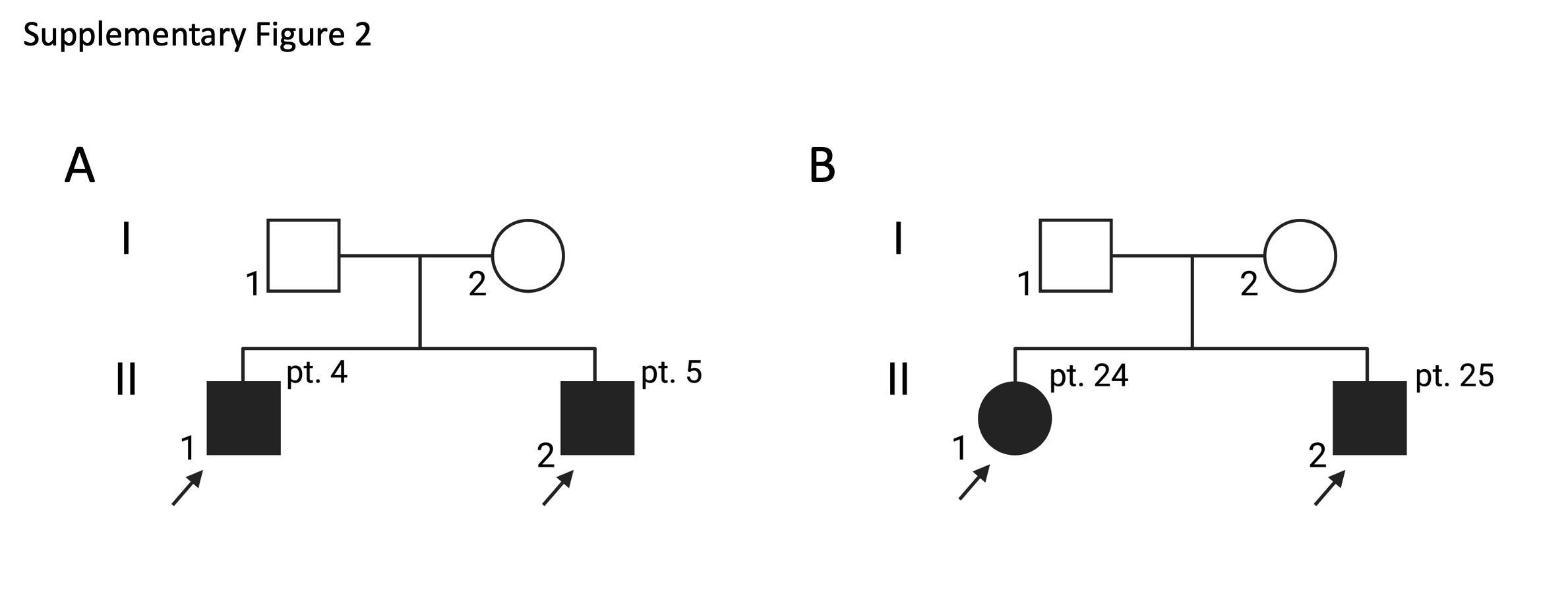

Supplement: Supplementary file 2 [file Image2.jpeg]
